# Does Radioactive Iodine Treatment Affect Thyroid Size and Tracheal Diameter?

**DOI:** 10.3390/jcm14010115

**Published:** 2024-12-28

**Authors:** Kadriye Yazici Demir, Zulkuf Kaya, Ramazan Dayanan, Tolga Mercantepe, Filiz Mercantepe

**Affiliations:** 1 Department of Otorhinolaryngology, Faculty of Medicine, University of Ataturk, Erzurum 25240, Türkiye; dr.kadriyeyazicidemir@kapadokyahastanesi.com.tr (K.Y.D.); zulkuf.kaya@atauni.edu.tr (Z.K.); 2 Department of Endocrinology and Metabolism, Batman Training and Research Hospital, Batman 72070, Türkiye; ramazan.dayanan@saglik.gov.tr; 3 Department of Histology, Faculty of Medicine, Recep Tayyip Erdogan University, Rize 53100, Türkiye; 4 Department of Endocrinology and Metabolism, Faculty of Medicine, Recep Tayyip Erdogan University, Rize 53100, Türkiye

**Keywords:** radioactive iodine, hyperthyroidism, tracheal diameter, thyroid size, Cavalieri method

## Abstract

**Background/Objectives:** There exist three principal treatment modalities employed in the management of hyperthyroidism attributable to excessive hormone secretion by the thyroid gland: antithyroid pharmacotherapy, surgical intervention, and radioactive iodine (RAI) therapy. Surgical intervention is typically indicated for markedly enlarged thyroid glands that exert pressure on the trachea. The objective of this investigation was to ascertain the influence of RAI on thyroid volume and tracheal diameter. **Methods:** This study included 20 patients, six females and 14 males, who received 20 mCi radioactive iodine treatment for toxic nodular goiter at a tertiary university hospital between March 2019 and February 2020. Pre-treatment and six-month post-treatment neck MRI scans were conducted on the cohort. Thyroid and tracheal volumes were quantified using the Cavalieri method based on MRI sections, and comparisons were conducted pre-and post-treatment. Statistical analysis of the comparative values was performed using the dependent samples *t*-test. **Results:** A statistically significant reduction in thyroid volume was observed among the 20 patients, averaging a decrease of 36.06% following RAI treatment compared to baseline measurements (*p* < 0.001). Additionally, an average increase of 12.76% in tracheal volume was noted post-treatment in comparison to initial measurements, which was also statistically significant (*p* < 0.05). None of the patients exhibited respiratory distress in the immediate postoperative period. **Conclusions:** The findings indicate that RAI therapy leads to a reduction in thyroid size, accompanied by an increase in tracheal diameters subsequent to treatment. Given the potential complications and risks associated with surgical intervention, it may be prudent to consider large thyroids for RAI therapy as an alternative to surgery.

## 1. Introduction

Hyperthyroidism represents a prevalent endocrine disorder, afflicting approximately 0.2 to 1.4% of the population globally, typically occurring between the ages of 20 and 40. This condition exhibits a higher prevalence in females, with a female-to-male ratio approximating 5:1 [1,2]. The predominant etiology of hyperthyroidism is Graves’ disease, which arises from an autoimmune mechanism [3]. In geographic areas characterized by iodine deficiency and among the geriatric population, toxic nodular goiter is encountered more frequently [4]. While three therapeutic modalities exist for the management of Graves’ disease—namely pharmacotherapy, surgical intervention, and radioactive iodine (RAI)—pharmacotherapy demonstrates limited efficacy in cases of toxic nodular goiter [5]. In such instances, RAI or surgical intervention is generally employed [6]. Antithyroid medications encompass compounds such as methimazole and propylthiouracil, which are extensively utilized in the management of Graves’ disease and toxic multinodular goiter. These pharmacological agents may elicit a spectrum of adverse effects, ranging from mild manifestations such as dermal reactions, alterations in taste perception, and arthralgia to more severe complications including agranulocytosis, hepatotoxicity, and ANCA-positive vasculitis [7,8]. Propylthiouracil is linked with the potential for hepatotoxicity and vasculitis, while methimazole is correlated with cholestatic adverse effects and teratogenicity [9,10]. Routine monitoring of hematological parameters and hepatic function tests is advised to mitigate the incidence of adverse effects [11].

Surgical intervention is regarded as a more efficacious approach for both thyrotoxicosis and symptoms related to compression. Nevertheless, it necessitates lifelong hormone replacement therapy, and there exists a risk for complications such as hypoparathyroidism and permanent recurrent laryngeal nerve paralysis [12]. I131 therapy is recognized as an effective and safe treatment modality in both primary management and secondary treatment in cases where a pharmacological response is inadequate [13,14]. In several European and Latin American nations, RAI therapy has supplanted surgical options in the management of hyperthyroidism [15], a transition that is also endorsed by contemporary clinical guidelines [16]. In scenarios involving hyperthyroidism with an enlarged thyroid gland, the prevailing clinical strategy is to recommend surgical intervention for the patient. This recommendation is predicated on the observation that RAI does not achieve sufficient volumetric reduction and may exacerbate compression symptoms through the potential induction of tracheal stenosis. The objective of this study was to elucidate the possible ramifications of RAI treatment on patients presenting with substantial thyroid volumes by assessing its impact on thyroid size and tracheal diameter utilizing the Cavalieri method, an objective measurement technique.

## 2. Participants and Methods

The present investigation received endorsement from the Ethics Committee at Atatürk University Faculty of Medicine (dated 13 March 2019 and numbered B.30.2.ATA.0.01.00/225). The research was executed in alignment with the principles outlined in the Declaration of Helsinki. Informed voluntary consent was duly acquired from every participant involved.

### 2.1. Study Design and Data Collections

The current investigation encompassed 20 patients who sought treatment at the Department of Endocrinology, Atatürk University Faculty of Medicine, and underwent 20 mCi radioactive iodine therapy due to toxic nodular goiter at the Department of Nuclear Medicine, Atatürk University Faculty of Medicine between March 2019 and February 2020. Prior to the administration of RAI, malignancy was systematically excluded in all participants. Magnetic Resonance Imaging (MRI) of the neck was conducted on the subjects both prior to and six months subsequent to the treatment. The peak efficacy of RAI treatment is typically observed within a timeframe of 4–8 weeks, with enhancements in thyroid function potentially persisting for as long as six months. Consequently, follow-up MRIs were executed on the subjects six months following the treatment. Thyroid and tracheal volumes were quantitatively assessed in the neck MRIs of the subjects utilizing the Cavalieri method, a recognized stereological approach, and comparative analyses were conducted before and after RAI. All calculations were performed by the same specialist on each occasion while remaining blinded to the treatment cohorts.

### 2.2. Calculation of Total Volume of Thyroid and Trachea

**Figure 1:**
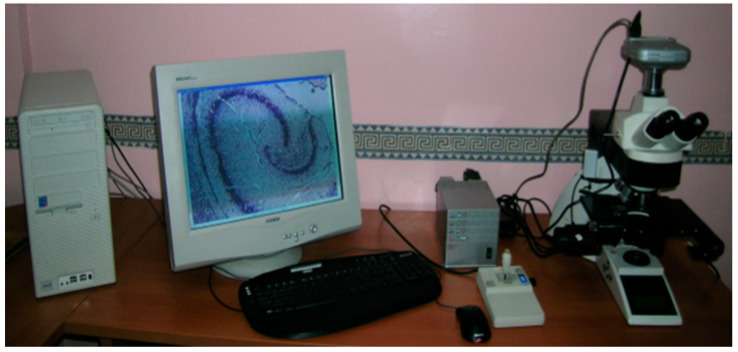
Stereo-Investigator (version 7.0, Microbrightfield, Colchester, VT, USA).

The Cavalieri method constitutes a non-invasive and highly accurate technique for volume measurement, grounded in fundamental stereological principles [17]. This methodology facilitates the estimation of the three-dimensional volume of any object by aggregating its two-dimensional cross-sectional areas [18]. Named in honor of the renowned Italian mathematician Bonaventura Cavalieri, this approach is extensively employed in the biomedical sciences for the accurate and reliable quantification of tissue volume or organ dimensions [18,19,20,21]. In the current investigation, a sophisticated instrument system known as Stereo-Investigator (version 7.0, Microbrightfield, Colchester, VT, USA) was employed, incorporating specialized software equipped with a dotted area ruler that facilitates area calculations via the Cavalieri method [18,19,20,21]. This instrument system comprises a microscope integrated with a digital camera, a motorized apparatus that facilitates the movement of the microscope stage, and a high-performance computer equipped with software that governs their operational parameters (Figure 1). In the context of this study, the computational component of this instrument system was exclusively utilized.

**Figure 2:**
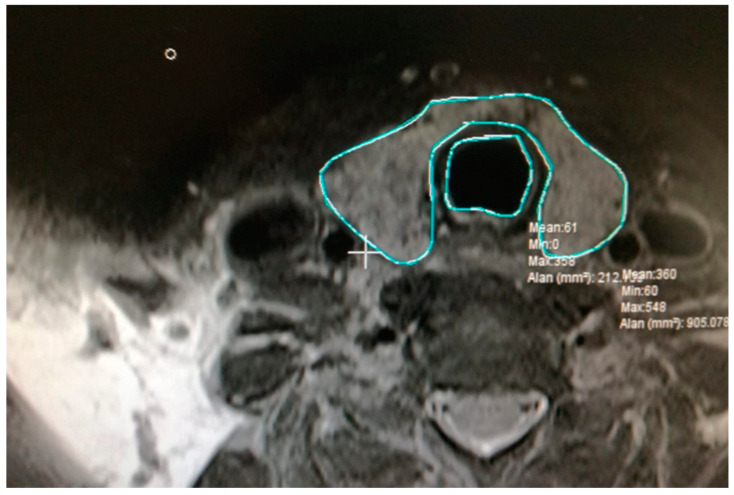
Marked image of the thyroid and trachea outlines in MRI slices.

**Figure 3:**
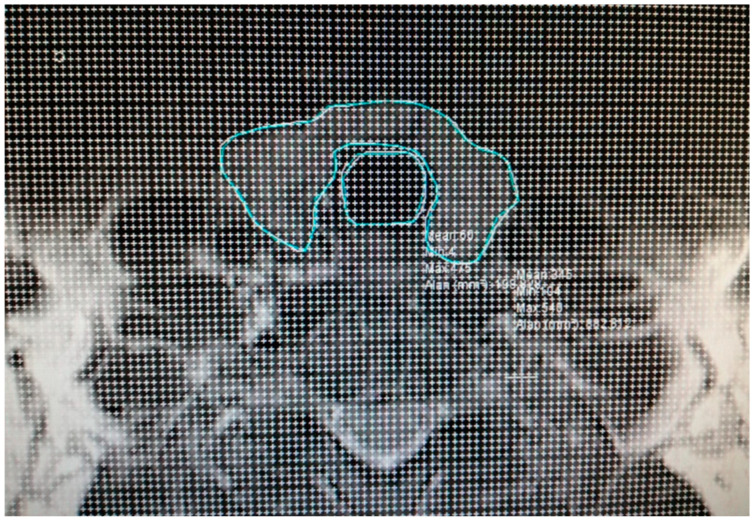
The dotted area ruler is placed on the section on the screen.

**Figure 4:**
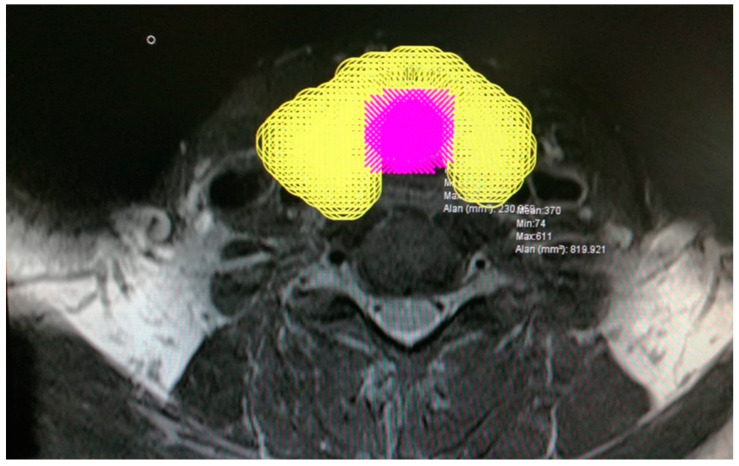
The points on the thyroid and trachea are marked with separate-colored markers.

All participants in the study underwent an MRI of the cervical region with 5 mm slice thickness both prior to and six months subsequent to RAI treatment. Thyroid sections within the MR images were meticulously selected for analysis. The delineation of the thyroid and trachea contours in the selected sections was performed (Figure 2). Subsequently, a dotted area ruler featuring a systematic arrangement of evenly spaced points was superimposed onto the section on the computer (Figure 3). The frequency of this dotted area ruler was determined in accordance with a suitable error coefficient value. In the subsequent phases of the study, the points corresponding to the thyroid and trachea were separately marked with distinct colored indicators (Figure 4).

According to the reference volume formula given below, in each section:

Reference V = t × a/p × Σ P

Expressions in the formula;

V: reference volume,

t: section thickness or distance between corresponding surfaces,

a/p is the area represented by a point,

Σ P: is the total number of points falling on the marked thyroid and trachea in a section.

The same procedures were applied for all sections containing the thyroid for all patients. Finally, in accordance with the Cavalieri principle, the software automatically calculated the total volume for the thyroid and trachea separately according to the following total volume formula [18,19,20,21].

Vtotal = V1 + V2 + … + Vn

Symbols of the formula:

Vtotal: total volume

V1: thyroid/trachea volume in the first section

V2: thyroid/trachea volume in the second section

Vn: thyroid/trachea volume in the last section

### 2.3. Statistical Analysis

Data were analyzed using Windows SPSS 20 package program. Pearson correlation coefficient was used to analyze the relationship between pre-treatment thyroid volume, the difference between pre- and post-treatment thyroid volumes, and the ratio parameters of pre- and post-treatment thyroid/trachea volume values. A comparison of pre- and post-treatment thyroid and trachea volume values was made with a dependent sample *t* test. A *p* value of less than 0.05 was considered statistically significant.

## 3. Results

The present study encompassed a cohort of 20 patients who underwent a 20 mCi RAI treatment for toxic multinodular goiter [22,23] during the period extending from March 2019 to February 2020. The data delineating the demographic characteristics of the patients, stratified by age and gender, are elucidated in Table 1. Upon examination of the gender distribution among the patients, it is observed that 6 (30%) were male and 14 (70%) were female. The predominance of female patients regarding thyroid disease aligns with the existing literature.

**Figure 5:**
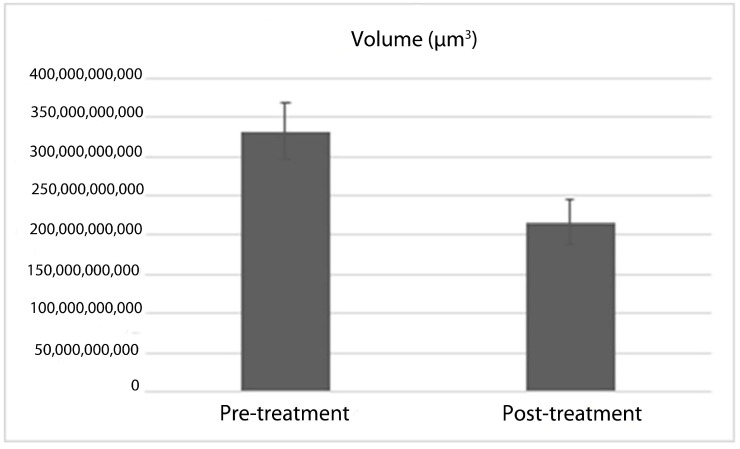
Pre- and post-treatment thyroid volume means.

The comparative analysis of thyroid and tracheal volumes among the participants, conducted prior to and subsequent to RAI treatment, is illustrated in Table 2. The results of the study revealed a statistically significant difference in the volume measurements of the thyroid and trachea pre- and post-treatment (*p* < 0.001, *p* < 0.05, respectively). When evaluating the thyroid volumes before and after treatment in terms of percentage change, it was found that the thyroid volume exhibited a reduction of 36.06% (Figure 5). The *p*-value determined through the dependent sample *t*-test was *p* < 0.001, indicating a statistically significant decrease. A little increase in volume was noted in three patients; however, this change did not reach statistical significance.

**Figure 6:**
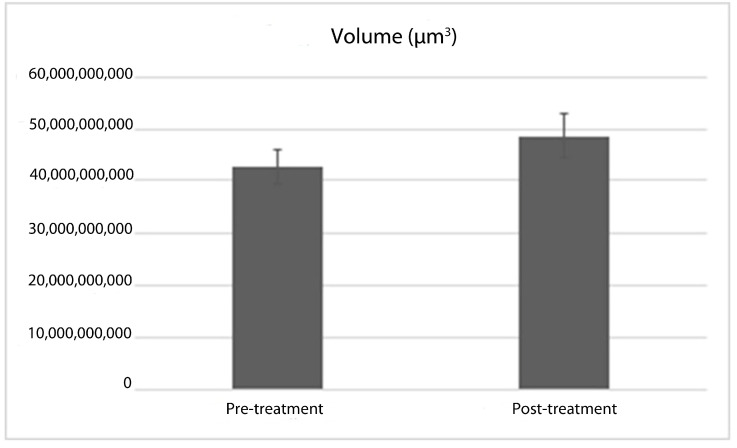
Pre- and post-treatment trachea volume means.

In the comparison of tracheal volumes before and after treatment, expressed as a percentage, it was observed that the tracheal volume increased by 12.76% (Figure 6). The *p*-value derived from the dependent sample *t*-test was *p* < 0.05, indicating a statistically significant increase. A reduction in tracheal volume was noted in four patients, while no volume alteration occurred in three patients. Nonetheless, these changes were statistically insignificant, and no instances of respiratory distress were recorded in the acute post-treatment phase among any of the patients.

**Figure 7:**
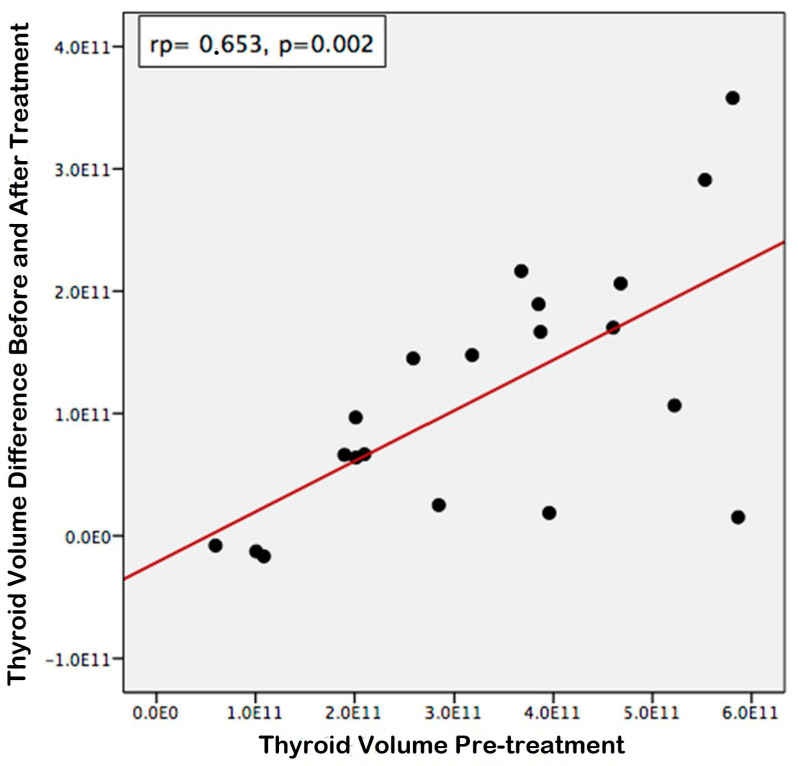
Correlation of pre-treatment thyroid volume and difference between pre- and post-treatment thyroid volumes.

**Figure 8:**
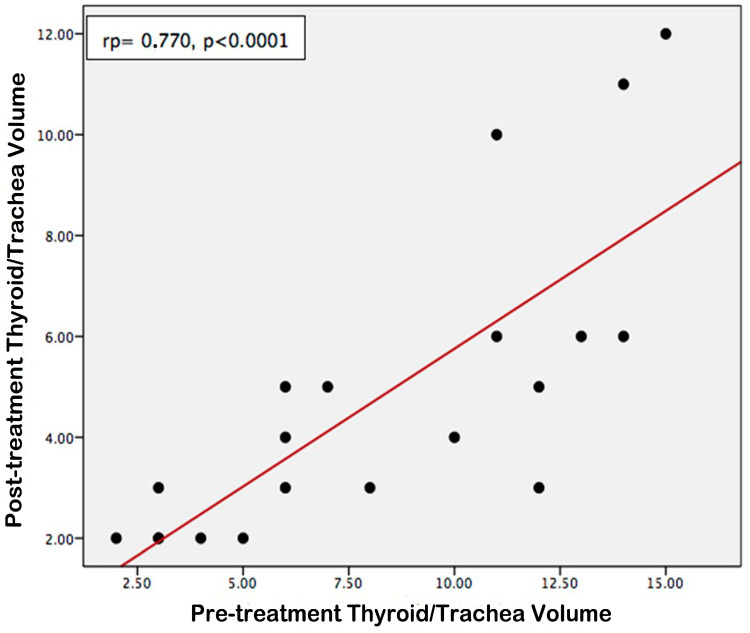
Correlation between pre-treatment thyroid/trachea volume ratio and post-treatment thyroid/trachea volume ratio.

According to the statistical evaluations conducted, a positive correlation was identified between the pre-treatment thyroid volume and the differential between the pre-treatment and post-treatment thyroid volumes (Figure 7, rp = 0.653, *p* = 0.002). Consequently, it was ascertained that larger pre-treatment thyroid volumes were associated with a more substantial decrease in volume following treatment. Furthermore, a positive correlation was established between the pre-treatment and post-treatment thyroid/tracheal volume ratios (Figure 8, rp = 0.770, *p* < 0.001). Thus, it was concluded that the ratios of pre-treatment thyroid/trachea volumes were directly proportional to those observed post-treatment.

## 4. Discussion

In the current investigation, thyroid volumes and tracheal diameters derived from MRI images obtained prior to and six months subsequent to the administration of treatment in 20 patients who underwent 20 mCi RAI therapy for hyperthyroidism were meticulously analyzed. Based on this analysis, it was determined that RAI treatment did not yield a statistically significant increase in thyroid volume; however, a notable increase in tracheal diameter was observed. This finding indicates that RAI treatment resulted in a reduction in thyroid diameter by approximately one-third when assessed after an extended duration, such as six months, and did not induce tracheal stenosis by causing an increase in tracheal diameter.

Hyperthyroidism represents a prevalent endocrine disorder within the population [24]. While some individuals with hyperthyroidism may remain asymptomatic, others may experience severe symptoms that markedly disrupt daily functioning [25]. Thyroid hormones serve as the primary regulators of basal metabolic rate. The most critical physiological effects of thyroid hormones include the elevation of heart rate, the enhancement of ventilation, and the reduction in peripheral resistance [26]. Furthermore, in the majority of hyperthyroid patients, the thyroid gland may hypertrophy to two to three times its standard size. The proliferation of cells is significantly augmented due to hyperplasia and follicular expansion. Additionally, the secretion rate per cell may increase several-fold [1].

As a consequence of the enlargement of the thyroid gland, certain patients may encounter cosmetic concerns, while others may suffer from respiratory distress attributable to the close anatomical relationship between the thyroid and trachea. Three principal modalities are employed in the management of hyperthyroidism: antithyroid drug therapy, surgical intervention, and RAI therapy [1,27]. The selection of the treatment modality is contingent upon the individual patient’s condition. There exists a lack of global consensus regarding the optimal initial treatment approach. Antithyroid medications are typically favored for the management of newly diagnosed hyperthyroid patients exhibiting mild symptoms [28]. Nevertheless, prolonged pharmacological therapy is generally discouraged due to the potential for adverse effects [11]. I-131 therapy has been utilized for over 70 years in the treatment of Graves’ disease and for 30 years in cases of benign nodular goiter. I-131 therapy not only addresses hyperthyroidism but also contributes to the reduction in thyroid volume [23,29,30,31,32]. Surgical intervention is predominantly indicated for the management of substantial goiters, as I-131 may be less efficacious and often necessitates a secondary RAI treatment [33,34,35]. Surgical options represent a more effective resolution for both thyrotoxicosis and compressive symptoms; however, they necessitate lifelong hormone replacement therapy and carry risks of complications such as hypoparathyroidism and permanent recurrent nerve paralysis [36]. In comparison, I-131 treatment is a more economical alternative to surgical intervention [37]. It can also be administered orally and offers the advantage of treatment in a single dose. I-131 therapy induces radiation exposure to thyroid follicle cells via beta particle radiation [32]. Consequently, cellular necrosis ensues, followed by inflammation and fibrosis. This cascade ultimately diminishes the capacity for hormone synthesis, thereby providing an efficacious treatment avenue for hyperthyroidism [38].

Particularly, patients diagnosed with toxic nodular goiter represent a cohort particularly well-suited for RAI therapy. In the context of toxic nodular goiter, the isotope I-131 demonstrates preferential accumulation in hyperfunctioning autonomous nodules, while the surrounding paranodular thyroid tissue experiences reduced exposure to radiation. Consequently, a significant proportion, specifically 80% or more, of individuals with toxic nodules attain a state of euthyroidism within a 12-month period, and the incidence of enduring permanent hypothyroidism is markedly lower in comparison with those with Graves’ disease [28]. The previous investigations into I-131 treatment have indicated that thyroid volume diminishes by 35–50% over the course of one year [39]. In the current study, a reduction in thyroid volume of 36.06% was noted six months post-treatment, aligning with the existing literature. A more pronounced decrease is anticipated with extended follow-up periods associated with RAI therapy. While individual responses to RAI treatment exhibit variability, there is often an amelioration of symptoms accompanied by a high level of patient satisfaction (63, 65, 67). Vereist et al. reported that optimal outcomes were typically observed within a timeframe of less than one year; however, the maximal therapeutic effect was reached between 24 and 30 months [40]. Bonnema et al. conducted measurements of thyroid volume via ultrasonography prior to, and at one year and three years subsequent to, I-131 treatment in a cohort of 43 patients. Their findings revealed that thyroid volume diminished by nearly 50% after one year, and no statistically significant difference was observed at the three-year mark compared to the one-year assessment [32]. Should symptoms of thyrotoxicosis persist due to inadequate volume reduction, a secondary I-131 treatment may be administered [41]. The existence of a substernal goiter does not negate the favorable outcomes associated with I-131 treatment [42]. Nonetheless, such goiters tend to be considerably large, and despite the administration of equivalent radiation doses, the relative reduction in goiter size resulting from I-131 treatment is inversely correlated with the initial goiter volume [23]. In contrast to the findings presented in this study, we observed that larger baseline thyroid volumes corresponded with greater reductions in thyroid volume. Massaro et al. conducted research involving 75 patients, measuring thyroid volumes via ultrasonography before and after I-131 treatment in subgroups comprising 27 patients with multinodular goiter (MNG), 32 with Graves’ disease, and 16 with uninodular goiter. They documented a volume reduction of 28.6% in the Graves’ cohort, 53.3% in the MNG group, and 57.8% in patients with uninodular goiter 12 months post-treatment [43]. The extent of thyroid volume reduction is contingent upon both the initial thyroid volume and the uptake of RAI [44]. In instances of significantly enlarged goiters, the insufficiency of adequate thyroid volume reduction constrains the application of I 131 therapies. Although the dosage of I 131 can be escalated to facilitate enhanced volume reduction, this adjustment concomitantly leads to an elevation in the radiation exposure experienced by the organism [22]. Furthermore, the efficacy of I 131 is diminished in goiters characterized by low radioactive iodine uptake (RAIU). Should the thyroid RAIU be augmented to enhance thyroid irradiation, the likelihood of successful I 131 interventions may be improved. Over the years, numerous strategies have been implemented, including the adoption of a low iodine diet, the administration of lithium, and the use of diuretics. Nevertheless, the most potent agent identified to date in this context is recombinant human TSH (rhTSH). RhTSH has been demonstrated to elevate 24 h RAIU by 100% or greater without impacting the half-life of I 131 [45,46,47,48].

Despite the long-standing application of I 131, it is not extensively favored for patients presenting with exceedingly large goiters. The rationale for this apprehension stems from the belief that the already enlarged thyroid gland may undergo further enlargement during the acute phase under the influence of I 131, potentially exacerbating tracheal compression [22,33]. Nonetheless, this anticipated complication has not manifested in clinical practice and remains undocumented [23]. In our investigation, no patient reported experiencing respiratory distress during the acute phase.

Numerous investigations within the existing literature have sought to ascertain the impact of RAI treatment on the diameter of the trachea. Bonnema et al. administered a high-dose mean of 61 mCi (ranging from 26 to 124 mCi) of I-131 to a cohort of 23 patients diagnosed with MNG whose thyroid volume exceeded 150 mL. A pre-treatment neck MRI was conducted on the subjects with 8 mm slices, followed by subsequent scans at 1 week and 1-year post-treatment. The dimensions of the thyroid and trachea were quantified in axial sections. Notably, the minimum cross-sectional area of the trachea exhibited a reduction of 9.2% within one week following treatment, while the tracheal area demonstrated a subsequent increase of 17.9% by the conclusion of the one-year period. Furthermore, it was documented that the thyroid volume diminished by 15% after one week and by 34% after one year. Importantly, no patient experienced acute respiratory distress during the treatment interval [23].

In our investigation, we observed that the tracheal volume expanded by 12.76% for six months subsequent to treatment. Albino et al. conducted neck MRI evaluations on 22 patients prior to I-131 treatment and at intervals of 2-, 7-, 180-, and 360 days post-treatment to assess tracheal diameter and thyroid volume. An indirect estimation of tracheal compression was achieved by analyzing the tracheal cross-sectional area (TCA). While no alterations in thyroid volume were noted on the 2nd and 7th days, a gradual decline was recorded at the 6th and 12th months. No statistically significant differences in TCA were identified following treatment [49].

The primary objective of our study was to elucidate the effects of RAI treatment on thyroid size and tracheal diameter employing an objective methodology. In our findings, it was determined that the trachea experienced an expansion of 12.76% and the thyroid volume demonstrated a decrease of 36.06% six months post-treatment. Acute tracheal compression and respiratory distress are frequently cited as justifications for discontinuing RAI treatment in cases of enlarged thyroids; however, acute respiratory distress did not manifest in any of the patients in the current study. Additionally, there exist scholarly articles in the literature that corroborate the absence of respiratory distress. In our cohort, the pressure subsided, and symptoms were alleviated six months following treatment. We posit that RAI treatment does not induce acute respiratory distress as previously apprehended and ultimately offers long-term relief to patients in a safe manner.

Our investigation undoubtedly possesses certain constraints, and these constraints should be regarded with prudence. Primarily, our investigation was characterized by a relatively modest sample size. Owing to the financial implications associated with MRI, longitudinal imaging could not be conducted on patients, particularly during the initial phase of post-RAI treatment. Nevertheless, patients were subjected to rigorous monitoring, particularly concerning the potential emergence of respiratory distress. Subsequent investigations that incorporate serial assessments of thyroid volume and tracheal diameters in a larger cohort and during the initial period will serve to mitigate these limitations.

## 5. Conclusions

The findings of the present investigation indicate that radioactive iodine therapy does not lead to an increase in thyroid volume, nor does it result in tracheal stenosis; consequently, patients may be appropriately referred for radioactive iodine therapy as an alternative to surgical intervention. Therefore, the likelihood of encountering surgical complications is mitigated. To thoroughly assess the initial effects of radioactive iodine treatment, it is imperative that future studies involve a larger cohort of patients and employ a standardized methodology for quantifying tracheal volume during the early stages of treatment.

## Figures and Tables

**Table 1:** Age and gender distribution of participants.

Variables		N = 20
Age, years (min-max)		61 (36–84)
Gender	Male, n (%)	6 (30)
	Female, n (%)	14 (70)

**Table 2:** Thyroid and tracheal volumes of participants pre- and post-RAI treatment.

Volume	Minimum Volume (µm^3^)	Maximum Volume (µm^3^)	Mean Volume (µm^3^)
Pre-RAI Thyroid volume	59,625,000,000	586,440,000,000	331,940,725,000
Post-RAI Thyroid volume	67,612,500,000	571,275,000,000	216,330,725,000
Pre-RAI Trachea volume	16,920,000,000	71,662,500,000	42,768,375,000
Post-RAI Trachea volume	17,010,000,000	99,450,000,000	48,594,875,000

## Data Availability

All data generated or analyzed during this study are included in this article. The data will be available upon reasonable request (contact persons: filiz.mercantepe@saglik.gov.tr).
